# Supramammillary projections to the lateral preoptic area regulate active behavior and dopamine release

**DOI:** 10.21203/rs.3.rs-11156760/v1

**Published:** 2026-10-28

**Authors:** Satoshi Ikemoto, Yosuke Arima, Zengyou Ye, Coleman Calva, Xia Min, John Gibbons, Jorge Mendoza, Beyonce Getachew, Sarah Johnson, Marziyeh Belal

**Affiliations:** National Institute on Drug Abuse

## Abstract

The supramammillary nucleus (SuM) promotes active behavioral responding by engaging ventral tegmental area (VTA) dopamine neurons that project to the nucleus accumbens (NAc), primarily via a glutamatergic SuM→medial septum (MS) pathway. However, the contribution of additional SuM projections to this process remains unclear. Here we combined anatomical tracing, fiber photometry, chemogenetic, and optogenetic approaches to investigate the SuM→lateral preoptic area (LPO) pathway in mice. SuM→LPO neurons possessed extensive collateral projections and were inhibited during consummatory behavior but activated by aversive and unexpected stimuli. Chemogenetic activation of these neurons reduced water intake and immobility during stress, whereas inhibition enhanced the appetitive phase of ingestive behavior and fear-related immobility. Optogenetic stimulation of SuM→LPO terminals was reinforcing, markedly increasing active behavioral responding, and the same stimulation elicited dopamine release in the NAc. These findings suggest the SuM→LPO pathway as a distinct circuit that, alongside the SuM→MS pathway, engages mesolimbic dopamine signaling to promote active behavioral responding to environmental challenges.

## INTRODUCTION

The supramammillary nucleus (SuM) plays a critical role in promoting active, approach-oriented behaviors in response to environmental challenges ^1^. SuM neurons respond robustly to salient events, including stress, novelty, and anticipation of food ^2-5^. Rather than mediating passive defensive behaviors such as freezing or grooming, activation of SuM neurons elicits vigorous, active responses such as jumping, digging, treading, and struggling under restraint ^6^.

These active responses may depend on the SuM's ability to engage the mesolimbic dopamine system ^7^. Stimulation of SuM neurons activates dopamine projections from the ventral tegmental area (VTA) to the nucleus accumbens (NAc) and reinforces instrumental approach behaviors ^7-11^. Collectively, these findings suggest that SuM networks mobilize active actions during threat or uncertainty by recruiting downstream mesolimbic dopamine pathways ^1^. One established pathway through which SuM neurons generate active actions involves glutamatergic SuM neurons projecting to the medial septum (MS), which then excite VTA dopamine neurons ^7^. Activation of SuM VGluT2 neurons projecting to the MS reinforces approach behavior and activates VTA→NAc dopamine neurons; SuM→MS glutamatergic neurons are thus currently considered a key route for regulating active coping responses. However, SuM neurons possess extensive collateral projections ^6,12,13^, raising the question of whether additional downstream targets also contribute to the coordination of active responses.

A major downstream target of SuM projections is the lateral preoptic area (LPO) ^14,15^. The LPO is strongly interconnected with the VTA and serves as a critical node for reward-seeking behaviors ^16-20^. LPO neurons are activated by aversive stimuli, and direct stimulation of the LPO is reinforcing and excites VTA dopamine neurons ^19^. These properties suggest that the LPO is a key component of neural circuits generating active responses to threat or uncertainty, with the SuM as a plausible upstream modulator. Recent work shows that SuM neurons projecting to the broader preoptic area are activated by acute stressors, and stimulation of this pathway promotes active responding during stressful conditions; however, activation of this circuit can also induce aversive or avoidance behavior ^6^. These findings raise the question of whether SuM-driven active responses depend solely on VTA→NAc dopamine neurons, or whether alternative SuM pathways can also generate active behaviors.

To clarify how SuM neurons communicate with downstream regions, the present study addressed four key questions. First, we mapped the extent to which SuM→MS and SuM→LPO neurons possess collateral projections, finding that both populations show extensive collateralization with substantial overlap. Second, we examined SuM→LPO activity during salient positive and negative stimuli, observing that these neurons are activated by threatening events and inhibited during ingestive behavior. Third, we investigated how manipulation of SuM→LPO+Collateral neurons affects ingestive, fear-related, and stress-coping behaviors, finding that activation enhances active coping responses while inhibition promotes passive responses and facilitates the appetitive phase of ingestive behavior. Finally, we tested whether selective SuM→LPO activation is reinforcing and triggers dopamine release, and found that such activation was reinforcing and induced dopamine release in the NAc. Together, these results demonstrate that SuM efferents have extensive collateral organization, enabling them to influence multiple downstream targets in parallel, and identify the SuM→LPO pathway as an anatomically distinct circuit—alongside the SuM→MS→VTA dopamine pathway—through which SuM neurons can generate active behaviors.

## RESULTS

For the rest of the paper, we refer to selective projections of SuM neurons to the LPO or the MS as SuM→LPO and SuM→MS, respectively. When we refer to SuM→LPO or SuM→MS neurons and their collateral projections, we use SuM→LPO+Collateral and SuM→MS+Collateral, respectively.

### SuM→LPO and SuM→MS neurons have collateral projections

To characterize SuM→LPO+Collateral and SuM→MS+Collateral, we injected retrograde AAV-Cre into either the LPO or MS and delivered Cre-dependent AAV-mGFP-2A-synaptophysin-mRuby into the SuM of wild-type mice (Fig. 1a). Both SuM→LPO and SuM→MS neurons exhibited extensive collateral projections throughout the brain and showed overall similar collateralization patterns (Fig. 1b-h; Table 1). SuM→LPO neurons appeared to possess more widely distributed collateral projections than SuM→MS neurons. Notably, SuM→LPO neurons showed stronger GFP expression in the lateral habenula (LHb), whereas SuM→MS neurons exhibited more pronounced GFP labeling in hippocampal CA1 and CA2 (Fig. 1f). Although both populations projected robustly throughout the septal area, which is adjacent to the NAc, neither population projected directly to the NAc.

To quantify overlap between SuM neurons projecting to the LPO and MS, we applied a dual tracing strategy in each mouse (N = 4): cholera toxin B (CTB) was injected into the MS and Fluoro-Gold (FG) into the LPO (Fig. 2a, b). CTB-labeled MS-projecting neurons were concentrated in the ventral SuM, extending from medial to lateral aspects (Fig. 2c). FG-labeled LPO-projecting neurons were located primarily in the medial SuM and extended dorsally and posteriorly into the VTA. Because the LPO tracer was delivered unilaterally, quantification was restricted to the ipsilateral SuM. Within a defined region of interest (ROI; 300 × 600 μm), dual labeling yielded 2,100 FG-positive and 1,259 CTB-positive neurons. Of these, 30% of LPO-projecting neurons were also CTB-positive, and 59% of MS-projecting neurons were also FG-positive (Fig. 2d). These findings indicate that most SuM→MS neurons collateralize to the LPO, whereas a smaller fraction of SuM→LPO neurons collateralize to the MS, supporting the conclusion that SuM→LPO neurons have more diverse downstream targets.

### SuM→LPO activity during salient positive and negative stimuli

To examine how SuM→LPO neurons respond to salient events, we expressed Cre-dependent GCaMP7s in SuM following retro-AAV-Cre delivery to the LPO and recorded SuM→LPO activity via fiber photometry (Fig. 3a, b). Activity was measured during water ingestive behavior, bright light, loud sound, and foot shock.

### SuM→LPO activity decreases during appetitive and consummatory phases of water intake

Trials were initiated by a nose poke at the start port (opNP), triggering a 2-s tone (Fig. 3c). The CS+ (20 kHz) predicted water delivery at the reward port (US+), available for consumption by nose-poking at the reward port (reNP). The CS− (3 kHz) predicted no water delivery (US−). GCaMP data were pooled across mice for sessions 1 and 9 (Fig. 3d-f), and reward and no-reward trials were analyzed separately.

For reward trials, a mixed ANOVA with epoch (baseline, opNP, CS+, reNP, US+) and session (1, 9) produced a significant epoch-by-session interaction, F(4, 3160) = 38.57, P < 0.001. In session 1, SuM→LPO activity decreased significantly during reNP and US+, but not during opNP or CS+ (Fig. 3e); in session 9, CS+, reNP, and US+ evoked even stronger suppression relative to both baseline and session 1. Thus, SuM→LPO neurons are suppressed during conditioned approach responses and consumption, and this suppression strengthens with training.

For no-reward trials, because mice performed reNP on ~ 60% of CS− trials, these trials were separated by whether reNP occurred. A three-way mixed ANOVA revealed a significant trial-type-by-epoch interaction, F(2, 1578) = 4.82, P = 0.008: CS− elicited significantly lower activity when followed by reNP compared with baseline and with CS− trials not followed by reNP (Fig. 3f). A second ANOVA found that reNP, but not the US− period, produced significant suppression across sessions. Overall, SuM→LPO neurons show strong decreases in activity during reward checking and consumption, and this suppression generalizes to conditioned stimuli predicting reward.

### SuM→LPO neurons increase activity in response to salient sensory stimuli, with stimulus-dependent adaptation

Mice received 100 trials of light or sound presented on a variable-interval (VI) 45-s schedule; GCaMP signals were analyzed for the 2 s before and after stimulus onset, with 10-trial bins forming blocks 1, 4, 7, and 10.

A 1-s bright light elicited strong time-locked increases in SuM→LPO activity (epoch effect: F(1, 15) = 48.45, P < 0.001) with no habituation across blocks (Fig. 3g). Loud noise also increased SuM→LPO signals, but with a significant epoch-by-block interaction, F(3, 42) = 16.38, P < 0.001 (Fig. 3h); responses in block 1 were significantly higher than in blocks 4, 7, and 10, while noise-induced signals were significantly greater than baseline signals at all trials.

Examining the first 21 trials revealed a significant epoch-by-trial interaction, F(4, 56) = 4.15, P < 0.005 (Fig. 3i), with responses declining significantly by trials 16 and 21. These findings indicate two components of SuM→LPO auditory responses: a rapidly habituating component and a sustained component that provides reliable responses throughout the session.

### SuM→LPO neurons increase activity in response to foot shock and conditioned stimuli

Foot shock and tone cues were presented in pseudorandom order (Fig. 3j). A mixed ANOVA revealed a significant condition-by-epoch-by-trial interaction, F(6, 90) = 2.31, P = 0.040 (Fig. 3k, l); foot shock reliably and robustly increased SuM→LPO activity across trials.

CS+ rapidly acquired the capacity to activate SuM→LPO neurons: responses were absent on the first presentation but significant on presentations 2–4. CS− increased activity early in training, likely reflecting tone generalization due to CS+ trials preceding CS−, but by trial 7 no longer increased SuM→LPO activity, indicating successful discrimination. Thus, SuM→LPO neurons show strong unconditioned responses to shock and rapid, learning-dependent increases to shock-predictive cues.

### Activation and inhibition of SuM→LPO+Collateral neurons alter motivated behaviors

We bilaterally expressed M3Dq, M4Di, or control mCherry in SuM→LPO+Collateral neurons to assess pathway-wide excitation or inhibition (Fig. 4a, b). Because SuM→LPO+Collateral neurons show opposite responses to ingestive versus threat-related stimuli, we examined behaviors across both domains.

### Novel open-field activity

Locomotor behavior was measured following CNO injection. A mixed ANOVA showed a significant group effect (F(2, 45) = 3.79, P = 0.030): M3Dq mice traveled significantly farther than M4Di mice, though neither differed from controls (Fig. 4c). Time in the center also showed a significant group effect (F(2, 45) = 3.90, P = 0.028), with M3Dq mice spending significantly less time in the center than controls, suggesting an anxiogenic effect of SuM→LPO+Collateral activation.

### Activation reduces water intake

Because SuM→LPO neurons are suppressed during consumption, we hypothesized that M3Dq activation would disrupt drinking. A significant group-by-session interaction emerged (F(2, 69) = 13.17, P < 0.001; Fig. 4d): CNO markedly reduced intake in M3Dq mice relative to saline and controls, whereas M4Di mice showed no effect.

### Inhibition enhances conditioned approach; activation disrupts it

Mice underwent Pavlovian conditioning in which a 6-s light predicted water delivery. A mixed ANOVA found significant group effect (F(2, 69) = 9.83, P < 0.001) and session-by-group effect (F(12, 414) = 1.86, P = 0.038);. During acquisition, M4Di mice showed higher approach probabilities than controls, whereas M3Dq mice showed reduced approach, especially early (Fig. 4e). Differences persisted in extinction session 8 but disappeared in session 9. Thus, inhibition of SuM→LPO+Collateral enhances appetitive responding, whereas activation disrupts it.

### Activation or inhibition does not produce conditioned place preference or avoidance

Because SuM→LPO neurons collateralize extensively, we anticipated that these manipulations might not produce clear conditioned place preference (CPP) effects. During conditioning, mice received saline in the morning (confined to one compartment) and CNO or methamphetamine in the afternoon (confined to the opposite compartment). Methamphetamine, used as a positive control, produced significant place preference (*t*(3) = 3.58, *P* = 0.037), whereas CNO manipulation of SuM→LPO+Collateral produced neither preference nor avoidance in any group (Fig. 4f).

### Inhibition enhances fear conditioning

Mice received CNO before conditioning, during which a white-noise CS was paired with footshock. On the test day (no CNO), the mice returned to the same chamber, and the groups did not differ during the initial context-only phase, but immobility differed during the context + CS phase, with a significant group-by-block effect, *F*(5.1, 113.6) = 2.46, *P* = 0.0365: M4Di mice showed significantly greater immobility in block 4 than M3Dq mice (Fig. 4g). To assess whether altered pain perception could account for this effect, we tested nociception on a hot plate; fentanyl significantly increased response latency, but neither M3Dq nor M4Di manipulation had a detectable effect (Supplementary Fig. 1). These findings suggest that SuM→LPO+Collateral inhibition enhances associative learning between CS and shock, whereas activation may weaken it.

### Activation and inhibition bidirectionally regulate immobility in the tail-suspension test

Mice received CNO and were suspended by the tail for 6 min. A mixed ANOVA revealed a significant group-by-block effect (*F*(22, 231) = 1.64, *P* = 0.039; Fig. 4h): M4Di mice entered an immobility-dominant phase (≥ 7–8 s of immobility per 30-s bin) ~ 30 s earlier than controls, whereas M3Dq mice maintained lower immobility throughout. Thus, SuM→LPO+Collateral activity regulates coping responses during inescapable stress, with activation reducing immobility and inhibition increasing it.

### Selective SuM→LPO activation is reinforcing and drives NAc dopamine release

To determine whether selective SuM→LPO activation is reinforcing, VGluT2-Cre mice received Cre-dependent ChR2, NpHR, or EYFP AAV vectors in SuM and an optic fiber above the LPO and underwent optogenetic intracranial self-stimulation (ICSS) (Fig. 5a-c). During acquisition (sessions 3–7), ChR2 mice earned significantly more photostimulation than control or NpHR mice (*F*(2.2, 26) = 21.97, *P* < 0.001) (Fig. 5d). Lever-press analysis showed a significant session-by-lever effect (*F*(9, 270) = 18.71, *P* < 0.001) with active-lever responses increased from sessions 2 to 3 and decreased from sessions 7 to 8 and again increased from sessions 9 to 10, while inactive-lever responses stayed stable (Fig. 5e). These results demonstrate that SuM→LPO activation is positively reinforcing.

To test whether SuM→LPO stimulation drives mesolimbic dopamine release, we recorded DA3m signals in the NAc during optogenetic stimulation of SuM→LPO terminals (Fig. 6a, b). Photostimulation produced a robust, time-locked increase in DA3m fluorescence in Chrimson-expressing mice but not controls (group × epoch: *F*(1, 6) = 25.22, *P* = 0.002; Fig. 6c, d). Thus, SuM→LPO activation rapidly drives dopamine release in the NAc.

## DISCUSSION

The present study shows that the SuM has a structural organization capable of exerting widespread influence over many regions through extensive collateral projections, and identifies the SuM→LPO pathway and its collaterals as circuitry through which SuM neurons can influence VTA→NAc dopamine neurons and modulate active, approach-oriented behavior. These findings indicate that SuM→LPO projections constitute an additional efferent route—alongside the SuM→MS pathway—that can influence VTA→NAc dopamine signaling and support active behavioral states.

Our anatomical experiments demonstrate that both SuM→LPO and SuM→MS neurons possess extensive collateral projections throughout the forebrain and midbrain, with overall similar collateralization patterns. Some differences were apparent: SuM→LPO collaterals were slightly more voluminous and diverse than SuM→MS collaterals, SuM→LPO neurons showed particularly strong innervation of the lateral habenula relative to SuM→MS neurons, and SuM→MS neurons showed stronger hippocampal projections. Dual-labeling experiments further revealed substantial overlap between the two projection-defined populations, with most SuM→MS neurons collateralizing to the LPO but only a smaller fraction of SuM→LPO neurons collateralizing to the MS. These results indicate that information transmitted from the SuM to either the LPO or the MS is concurrently broadcast to multiple additional downstream nodes, enabling SuM neurons to coordinate distributed circuits involved in arousal, affect, and motor activation.

Fiber photometry recordings revealed a functional organization of SuM→LPO activity across motivational states. SuM→LPO neurons were strongly inhibited during the consummatory phase of water ingestion, replicating our previous finding that SuM neuron activity decrease during sucrose consumption ^7^ or food ^6^, and extending it by showing that SuM→LPO neurons were also inhibited during appetitive response to reward, with suppression strengthening across training. This indicates that SuM→LPO neurons are not simply responsive to all salient events; rather, they are specifically suppressed during appetitive and consummatory ingestion—states that require internal engagement rather than external vigilance or action. Suppression during CS− trials in which mice checked the reward port further supports the interpretation that this signal reflects the behavioral state of ingestion rather than reward value per se. In contrast, SuM→LPO neurons increased activity robustly in response to threats and threat-predictive cues, including unusual sensory events. Both bright light and loud noise increased SuM→LPO activity, though with different temporal dynamics: bright light elicited sustained increases without habituation, whereas loud noise produced an initial strong response that partially adapted over trials, suggesting a modality-selective habituation process and the coexistence of rapidly habituating and persistent auditory-response components. SuM→LPO neurons also showed strong unconditioned responses to foot shock and rapidly acquired responses to shock-predictive cues, with CS+ responses emerging within a few trials and CS− responses declining once mice discriminated the two tones. Together, these findings suggest that SuM→LPO neurons respond to unconditioned stimuli and rapidly acquire the associative significance of new stimuli, with their activity level modulated by external challenges.

Chemogenetic manipulations reveal that SuM→LPO+Collateral neurons exert opposing influences on active versus passive responses across multiple behavioral contexts. Activation of these neurons (M3Dq) increased locomotor activity and reduced immobility in the tail suspension test, consistent with promoting active coping during stress, whereas inhibition (M4Di) accelerated the onset of immobility during inescapable tail suspension and the acquisition of conditioned fear response (footshock conditioned immobility), indicating that reduced SuM output facilitates the expression and acquisition of passive responding under threat. Activation of SuM→LPO+Collateral neurons also suppressed water intake and disrupted conditioned approach to water, whereas inhibition facilitated approach and strengthened the conditioned appetitive response, with little or no effect on the consummatory response itself. Together with the photometry findings, these results suggest that SuM→LPO neurons are inhibited during ingestive behaviors, and that their activation can interfere with ingestive behavior.

Chemogenetic manipulations did not produce conditioned place preference or avoidance. It remains unclear whether SuM→LPO+Collateral neurons contribute to affective valence at all, or whether opposing effects transmitted through multiple collateral pathways cancel out any net preference or avoidance. Prior work shows that selective stimulation of the SuM→MS pathway produces real-time place preference, whereas selective stimulation of SuM neurons projecting to the paraventricular thalamic nucleus produces real-time place avoidance ^7^, raising the possibility that manipulations of SuM→LPO+Collateral neurons produced similarly mixed affective effects. Given that SuM→LPO neurons send collateral projections to the lateral habenula, an established aversive hub ^21-23^, M3Dq activation may have partly recruited the SuM→LHb pathway; consistent with this, our open-field data suggest a mildly anxiogenic effect of M3Dq activation, implying an aversive component to this manipulation. Moreover, Escobedo and colleagues ^6^ reported that continuous photostimulation at 1–20 Hz of ChR2-expressing SuM→LPO+Collateral neurons induces real-time place avoidance, further supporting the possibility of mixed valence within this pathway.

Although broad SuM→LPO+Collateral activation did not produce place preference, selective optogenetic stimulation of SuM terminals in the LPO was reinforcing in an intracranial self-stimulation task, and this stimulation produced time-locked dopamine release in the NAc. These results identify SuM→LPO projections as a pathway capable of modulating dopamine release, in addition to the previously characterized SuM→MS→VTA circuit. Taken together with the photometry findings, our results suggest that SuM neurons can mobilize active behavioral states through more than one downstream route: the SuM→MS circuit provides one established mechanism for driving VTA dopamine, while the SuM→LPO pathway represents a second circuit capable of reinforcing approach behavior and modulating dopamine release.

Several limitations should be noted. The precise contribution of parallel SuM projections to active responses remains unresolved. Fiber photometry detects population-level activity rather than single-unit activity, and the chemogenetic manipulations in this study targeted SuM→LPO neurons non-selectively with respect to their extensive collateral projections. Future work examining the activity and function of SuM neurons with respect to individual, selectively defined projection populations will be needed to resolve the distinct contributions of parallel efferent pathways.

In conclusion, this study demonstrates extensive collateral organization of SuM neurons, indicating that SuM neurons engage multiple downstream structures in parallel to meet external challenges. We further show that SuM→LPO+Collateral neurons modulate active responses to external challenges, and identify the SuM→LPO pathway as a downstream route that can influence active, approach-oriented behavior, functioning alongside the SuM→MS pathway in modulating mesolimbic dopamine. These findings broaden the understanding of supramammillary function and establish the LPO as a node through which SuM neurons regulate behavioral mode, salience processing, and reinforcement.

### Stereotaxic surgery

## METHODS

### Animals

C57BL/6J mice were purchased from the Jackson Laboratory. VGluT2-Cre mice (*Slc17a6*^tm2(cre)Lowl^/J) on a C57BL/6J background were bred at the National Institute on Drug Abuse (NIDA) Transgenic Breeding Facility. Both male and female mice were used in all experiments. Mice were 2–4 months old and weighed 25–35 g at the time of surgery. When not undergoing behavioral testing, mice were group-housed in a temperature- and humidity-controlled vivarium (70–74°F; 35–55% humidity) under a reversed 12:12 h light–dark cycle with lights off at 07:00. Food and water were available *ad libitum*, except for mice assigned to water-restriction experiments. For experiments using water as a reward, mice were placed under moderate water restriction for at least 48 h before behavioral testing to enhance water-seeking behavior; during restriction, mice were given access to water for 10 min per day, typically after behavioral testing, and body weight was monitored daily.

### Viral vectors, tracers, and pharmacological agents

All AAVs were purchased from Addgene, except DA3m, which was sourced from Biohipp (Table 2). AAV titers were approximately 1.0 × 10^13 genome copies/ml. Fluoro-Gold (Fluorochrome) and cholera toxin subunit B (CTB; List Labs) were diluted to 1% and 0.5%, respectively, in filtered water. Fentanyl and methamphetamine were obtained from the NIDA Drug Supply Program. Fentanyl (200 μg/kg), methamphetamine (1 mg/kg), and clozapine-N-oxide dihydrochloride (CNO; 3 mg/kg; Hello Bio) were dissolved in 0.9% saline (10 ml/kg) and administered intraperitoneally.

Mice were anesthetized with isoflurane (1–2%) and placed in a stereotaxic apparatus. Viral vectors or tracers were microinjected through a 34-gauge beveled needle using a syringe pump (Micro 4, World Precision Instruments) at 50 nl/min; the needle was left in place for an additional 10 min after each injection before withdrawal. Coordinates for each targeted brain region are listed in Table 3, given in millimeters relative to bregma and the skull surface unless otherwise specified. For fiber photometry and optogenetics, optic fibers were implanted above the target site and secured to the skull with dental cement. For fiber photometry, optic fibers (200 μm core diameter, 0.37 NA) were implanted 0.2 mm above the LPO and NAc and secured with Geristor A and B cement (DenMat; part #4506 and #034522101). For optogenetic behavioral experiments, a 200-μm, 0.39-NA optic fiber was implanted above the right LPO. Mice were allowed 4–5 weeks for viral expression before behavioral or photometry experiments. For chemogenetic experiments, a retrograde AAV-Cre vector was injected bilaterally into the LPO, and a single injection of an inhibitory (M4Di, Gi-coupled) or excitatory (M3Dq, Gq-coupled) DREADD or control vector was delivered to the midline SuM.

### Histology and imaging

Mice were deeply anesthetized and transcardially perfused with ice-cold 1× PBS followed by 10% formalin. Brains were post-fixed as appropriate, cryoprotected in 20% sucrose in PBS for 1–2 days, frozen, and coronally sectioned at 40 μm. For retrograde and viral tracing experiments, mice were perfused 1 week after tracer injection or 5 weeks after viral injection, as specified in the relevant sections below. For fluorescence immunostaining, sections were incubated in blocking buffer (PBS with 0.3% Triton X-100 and 1.5% normal donkey serum) for 30 min, followed by overnight incubation with primary antibodies diluted in blocking buffer, washing, and 2-h incubation with secondary antibodies. Sections were mounted with ProLong Diamond mounting medium (Thermo Fisher Scientific). For retrograde tracing experiments, CTB and Fluoro-Gold were detected by immunostaining using goat anti-CTB antibody (1:5000; List Biological Laboratories) and rabbit anti-Fluoro-Gold antibody (1:100; MilliporeSigma), followed by donkey anti-rabbit Alexa Fluor 488 (1:1000) and donkey anti-goat Cy3 (1:1000; Jackson ImmunoResearch) secondary antibodies. For collateral-projection, fiber photometry, and DREADD experiments, Cre was detected using mouse anti-Cre primary antibody (1:1000; MilliporeSigma) and donkey anti-mouse Cy5 (collateral-projection and photometry experiments) or Alexa Fluor 488 (DREADD experiments) secondary antibody (1:1000; Jackson ImmunoResearch). Low-magnification images were acquired with a BZ-X710 microscope (Keyence); high-magnification images were acquired with an FV1000 (Olympus) or Zeiss LSM880 confocal microscope, as appropriate. Injection sites, viral expression, tracer deposition, optic-fiber placement, and fluorescent labeling were verified histologically, and animals with mistargeted injections or misplaced fibers were excluded according to predefined anatomical criteria.

### Fiber photometry

A fiber photometry system (Doric Lenses) with a dual-wavelength LED driver was used. Excitation light consisted of sinusoidal waveforms at 465 nm (531 Hz) for GCaMP and 405 nm (208 Hz) for the isosbestic control. Light was transmitted through a fluorescence mini-cube and patch cables (0.48 NA, 400 μm core) to the implanted optic fiber (0.37 NA, 200 μm core). Emitted fluorescence (465 nm for GCaMP and DA3m, 405 nm for the control channel) was collected through the same fiber assembly and detected by photoreceivers via bandpass filters and beamsplitters. Signals were acquired at 1,200 Hz using Doric Neuroscience Studio software and synchronized with stimulation or behavioral events. Data were analyzed using custom Python scripts; GCaMP, DA3m, and control-channel signals were binned into 1-min epochs, the control channel was linearly fitted to the GCaMP or DA3m signal, and ΔF/F was calculated as ΔF/F = (F_signal - F_fitted control) / F_fitted control.

### Collateral projections of SuM→MS and SuM→LPO neurons

To examine collateral projections of MS− and LPO-projecting SuM neurons, we injected AAV-retro-EF1a-Cre into the MS or LPO and AAV1-FLEX-mGFP-2A-SYP-mRuby into the SuM of C57BL/6J mice, with vectors infused at 50 nl/min as described above. Five weeks after surgery, mice were perfused and brains processed for fluorescence imaging; mGFP signal identified Cre-dependent somatic and axonal labeling, and SYP-mRuby signal was used to evaluate putative synaptic terminal labeling in downstream regions. Low-magnification images were acquired with the BZ-X710 (Keyence). A total of 9 C57BL/6J mice (4 male, 5 female) were used for this experiment.

### Ratio of collateral projections between MS− and LPO-projecting SuM neurons

To visualize SuM neurons projecting to the MS, LPO, or both targets, we injected 1% Fluoro-Gold (100 nl; Fluorochrome) into the LPO and 0.5% CTB (150 nl; List Labs) into the MS of 4 C57BL/6J mice (1 male, 3 female). One week after tracer injection, mice were perfused and brain sections processed for immunofluorescence staining and imaging. SuM neurons containing FG, CTB, or both tracers were identified across anatomically matched sections. Colocalization analysis was performed using Huygens Suite (Scientific Volume Imaging). Rectangular ROIs (300 × 600 μm) were placed in the right SuM across three coronal sections representing anterior, middle, and posterior SuM levels (approximately bregma − 2.7 to − 3.0 mm). Double-labeled neurons were counted as FG+/CTB+ cells, and counts were summarized as the number or percentage of single- and double-labeled neurons relative to the total number of retrogradely labeled SuM neurons.

### GCaMP signal recording in SuM neurons projecting to the LPO during salient events

To determine how SuM neurons that broadly project to the LPO and other motivation-related regions respond to threats, salient stimuli, and consumable rewards, we injected retro-AAV-Cre into the LPO and AAV-FLEX-GCaMP7s into the SuM and recorded activity in response to these stimuli. Fiber photometry recordings were performed using the system described above; signals were acquired using Doric Neuroscience Studio software and synchronized with behavioral event timestamps generated by Med Associates programs. For event-aligned analyses, ΔF/F signals were z-scored relative to a pre-event baseline, and mean event-aligned traces and area-under-the-curve (AUC) values were extracted within predefined analysis windows for each behavioral event. Recordings were performed during the salient events described below, in the order conducted.

#### Stimulus discrimination conditioning with water.

Water-restricted mice were placed in a conditioning chamber equipped with a house light, an auditory cue generator, an operant (initiation) nose-poke port, and a reward nose-poke port. During the first five sessions (magazine training), mice were habituated to make a nose-poke response at the reward port (reNP), which triggered a 1-s click sound and 5-μl water delivery; each session lasted 60 min. Following magazine training, mice were trained on a discrimination task: a nose-poke response at the initiation port (opNP) turned off the house light and, after a 0.5-s delay, triggered one of two 2-s tones — a 20-kHz tone (CS+) followed by 1-s activation of the reward syringe (with a brief click) beginning 1.7 s after CS+ onset, or a 3-kHz tone (CS−) with no consequence. The CS+/CS− assignment was reversed in 3 mice. Ten seconds after CS− onset, the house light was restored, signaling availability of the next trial (initiated by an opNP response). Each session lasted 60 min.

#### Salient-stimulus tests.

Mice were returned to *ad libitum* water access and placed in a pitch-dark chamber in which a 60-W lightbulb (840 lumens) was illuminated for 1 s on a VI 45-s schedule, repeated 100 times. In a separate session, a 1-s, 115-dB auditory stimulus (Tokatuker alarm siren) was presented 100 times on the same schedule. The order of light and sound sessions was reversed in 3 mice to counterbalance order effects.

#### Cue–footshock test.

Mice underwent cue discrimination conditioning with footshock in Med Associates chambers. On CS+ trials, a 5-kHz tone was presented for 2 s and a 0.3-s, 0.45-mA footshock delivered 1.7 s after CS+ onset; on CS− trials, a 15-kHz tone was presented for 2 s with no footshock. Trials were pseudorandomized and separated by a VI 60-s schedule; the CS+/CS− assignment was reversed in 3 mice.

### Chemogenetic manipulation of SuM neurons projecting to the LPO (and their collaterals) during motivated behavior

To determine whether chemogenetic activation or inhibition of SuM→LPO neurons alters behavioral responses, C57BL/6J mice received bilateral injections of a retrograde AAV-Cre vector into the LPO and a Cre-dependent M4Di(Gi)-mCherry, M3Dq(Gq)-mCherry, or mCherry control AAV into the SuM. CNO (3.0 mg/kg, i.p.) or saline (10 μl/g, i.p.) was administered 10–20 min before testing. Mice underwent the behavioral assays below in the order presented, with at least 24 h between assays.

#### Open-field test.

Mice received CNO and were placed in a rectangular open-field arena (40 × 40 × 40 cm) for 20 min of free exploration. The central zone was defined as a 23.8 × 23.8 cm square centered in the arena; the remaining area was defined as the peripheral zone. Activity was recorded and analyzed using EthoVision video-tracking software.

#### Water intake test.

Mice underwent the water-restriction regimen and were placed in the test chamber for two 30-min baseline sessions, during which each nose-poke at the reward port delivered 5 μl of water. Mice then received saline and CNO (one per session, in the same chamber) and were allowed to consume water freely for 30 min in each of two subsequent sessions.

#### Conditioned approach response for water.

Water-restricted mice underwent Pavlovian conditioning in which a 6-s CS (house light off, cue lights on at the reward port) predicted water delivery; 5 s after CS onset, 5 μl of water was delivered with a 1-s click. Mice received CNO before acquisition sessions 1–7 but not before extinction sessions 8–9. Each session consisted of 60 trials separated by a VI 60-s schedule.

#### Conditioned place preference.

After return to *ad libitum* water access, mice underwent conditioned place preference (CPP) testing in a three-compartment chamber. On day 1, mice freely explored all compartments for 15 min without CNO. Over the next 3 days, mice received saline in the morning (confined to one compartment) and CNO or methamphetamine (1 mg/kg, i.p.; positive control) in the afternoon (confined to the opposite compartment), 30 min per session. On the test day, mice were placed in the apparatus without injection and given free access to all compartments for 15 min. EthoVision XT tracked location to determine time spent in each compartment; place preference was defined as the change in time spent in the CNO-paired compartment between pre- and post-conditioning sessions.

#### Fear conditioning and conditioned fear test.

Fear conditioning was conducted in chambers equipped for footshock delivery and video monitoring. Mice received CNO before conditioning. After a 3-min baseline, each mouse received six pairings of a white-noise CS (70 dB, 6 s) and a footshock US (0.45 mA, 1 s), with the footshock delivered 5 s after CS onset and co-terminating with the CS; pairings were separated by a VI 60-s schedule. The following day, mice returned to the same chamber without CNO for a 7-min test session using the same CS but no footshock. During the first 3 min (no CS), freezing was assessed to measure context-driven fear; a 6-s CS was then presented on a VI 60-s schedule. EthoVision XT analyzed video footage, identifying each mouse by grayscale value changes and scoring immobility as periods with pixel fluctuation below a 0.02% threshold lasting at least 0.75 s.

#### Hot plate test.

Nociceptive responses were assessed on a hot plate apparatus (Ugo Basile) set to 53.5°C. After CNO administration, mice were placed on the hot plate and latency to the first nociceptive response (hind-paw withdrawal, hind-paw licking, vocalization, or jumping) was recorded, with a 30-s cutoff to prevent tissue damage ^24^. Fentanyl (200 μg/kg, i.p.) was administered as a positive analgesic control on a separate day.

#### Tail suspension test.

Tail suspension testing was performed to assess stress-coping behavior, adapted from ^25^. After receiving CNO, each mouse was suspended by the tail for a 6-min session in a custom tail-suspension box under continuous white noise (70–72 dB). While the mouse rested on a platform, a 12-cm strip of adhesive tape was attached to the distal tail and secured to a horizontal bar; a lightweight plastic tube (40 × 10 × 8.5 mm; ~0.9 g) was placed around the tail before suspension to reduce tail-climbing. The platform was then removed to begin the test. Sessions were video recorded, and mobility/immobility were scored using the iMOSS workflow; primary outcomes were total immobility time and mobility/immobility bout measures over the 6-min session.

### Intracranial self-stimulation

VGluT2-Cre mice received AAV9-DIO-ChR2-EYFP (*N* = 16), AAV9-DIO-EYFP (*N* = 7), or AAV9-DIO-eNpHR3.0-EYFP (*N* = 6) into the SuM, with an optic fiber above the right LPO. ICSS was conducted in standard operant chambers with two levers and cue lights. During the first two sessions, an active-lever press triggered a 1-s cue light with no photostimulation. During acquisition sessions 3–7, an active-lever press triggered the cue light together with an 8-pulse, 25-Hz train of 473-nm photostimulation for the ChR2 and EYFP groups or 3-s continuous 575-nm photostimulation for the NpHR group. During extinction sessions 8–9, the cue-light contingency was maintained but photostimulation was withheld. In session 10, photostimulation was reinstated using the same contingencies as during acquisition. Each session lasted 30 min.

### Fiber photometry procedure to detect dopamine release

Mice received AAV5-FLEX-ChrimsonR or a AAV9-DIO-mCherry into the SuM, together with AAV5-GRAB-DA3m into the NAc, with an optic fiber above the LPO (for stimulation) and another above the NAc (for recording). NAc dopamine signals were recorded while an 8-pulse, 25-Hz train of 638-nm red laser light (10 mW, 3-ms pulse width) was delivered to the LPO on a VI 45-s schedule. Fiber photometry recordings and data analysis were performed as described above.

### Data analysis and statistics

Behavioral, histological, and photometry data were analyzed using custom Python scripts, GraphPad Prism, IBM SPSS Statistics, and other experiment-specific software. For histological tracing, labeled neurons or fluorescence signals were quantified within predefined anatomical ROIs. For behavioral assays, the animal was used as the statistical unit unless otherwise specified. For photometry experiments, fluorescence traces were aligned to behavioral or optical-stimulation events, normalized to a pre-event baseline, and summarized as event-aligned traces and AUC values within predefined time windows. Data are presented as mean ± SEM unless otherwise specified. Table 4 summarizes experimental designs and statistical tests used for data analyses. ANOVAs were followed by appropriate *post hoc* comparisons when justified. Statistical significance was set at *P* < 0.05.

## Supplementary Material

This is a list of supplementary files associated with this preprint. Click to download.

• SUPPLEMENTARYINFORMATION.docx

## Figures and Tables

**Figure 1:**
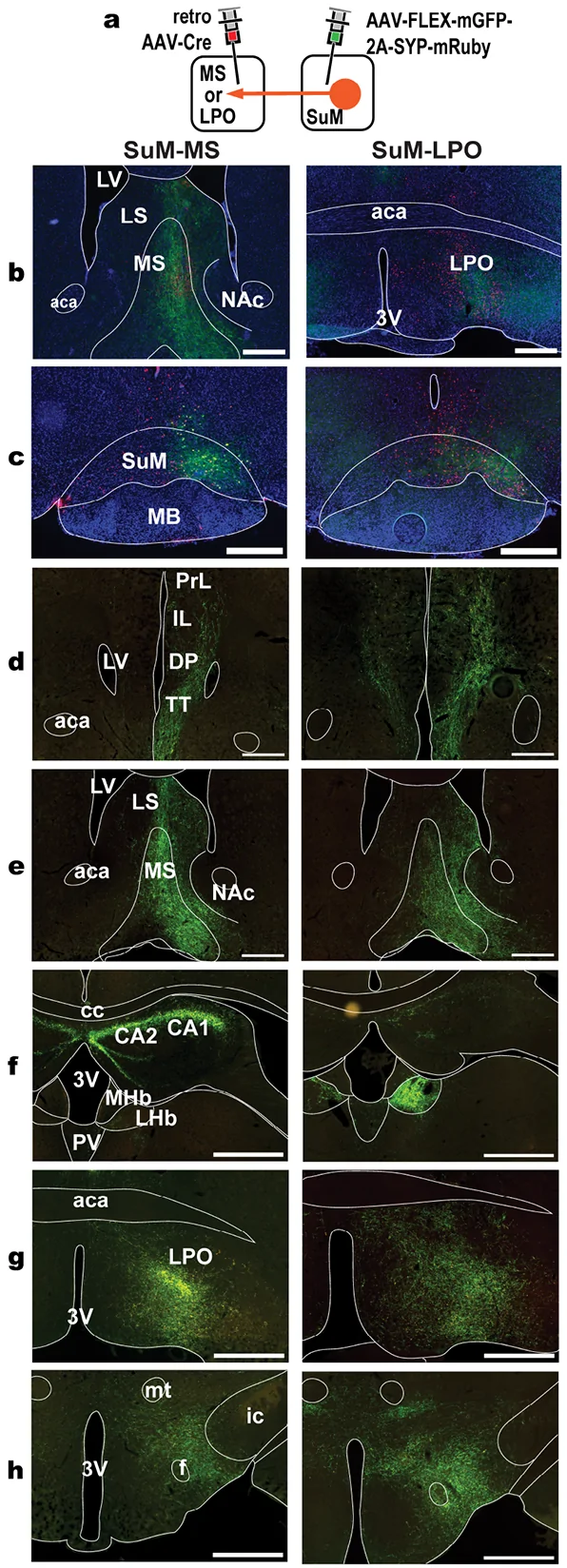
Collateral projections of MS− and LPO-projecting SuM neurons. (a) Experimental design. (b–c) Retrogradely Cre-labeled SuM neurons projecting to the MS (N = 5) or LPO (N = 4) (red), with mGFP-labeled SuM neurons indicating collateralized axon origins. (d–h) Collateral projection patterns of SuM→MS and SuM→LPO neurons across the medial prefrontal cortex (d), septal region (e), hippocampal and epithalamic regions (f), preoptic region (g), and hypothalamus (h). Synaptophysin-mRuby–positive terminals were confirmed at higher magnification. Scale bars, 500 μm. Abbreviations: 3V, third ventricle; aca, anterior commissure (anterior); CA1/CA2, hippocampal regions; cc, corpus callosum; DP, dorsal peduncular area; f, fornix; ic, internal capsule; IL, infralimbic cortex; LHb, lateral habenula; LPO, lateral preoptic area; LS, lateral septum; LV, lateral ventricle; MB, mammillary body; MHb, medial habenula; MS, medial septum; mt, mammillothalamic tract; NAc, nucleus accumbens; och, optic chiasm; PrL, prelimbic cortex; PV, paraventricular thalamic nucleus; SuM, supramammillary nucleus; TT, tenia tecta.

**Figure 2:**
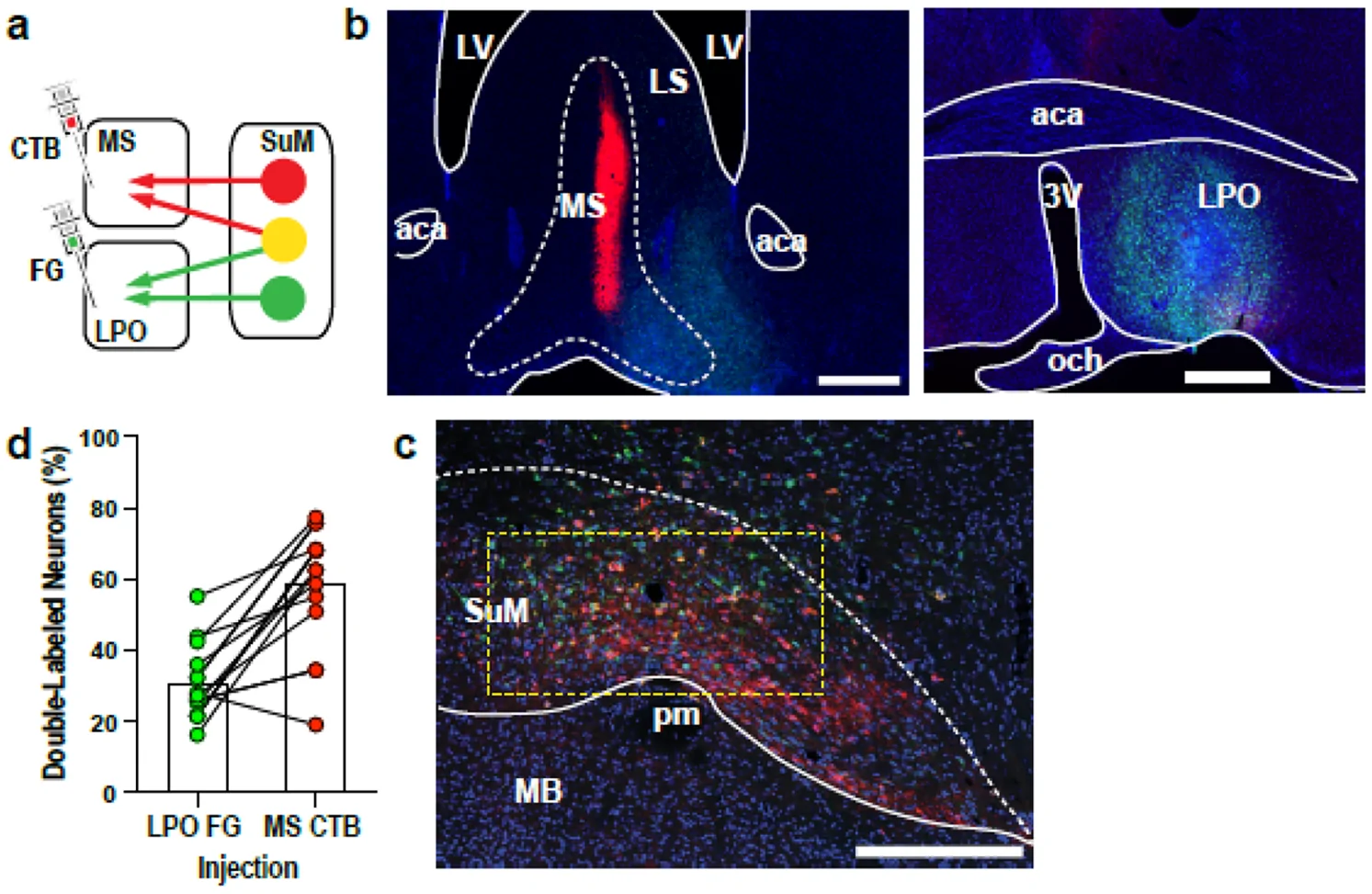
Overlap between SuM neurons projecting to the LPO and MS. (a) Schematic of dual retrograde tracer injections into the MS (CTB) and LPO (FG). (b) Representative injection sites. Scale bars, 500 μm. (c) Retrogradely labeled SuM neurons projecting to the MS (red), LPO (green), or both (orange). The outlined ROI indicates the quantified region. (d) Percentage of double-labeled neurons. N = 4 mice.

**Figure 3:**
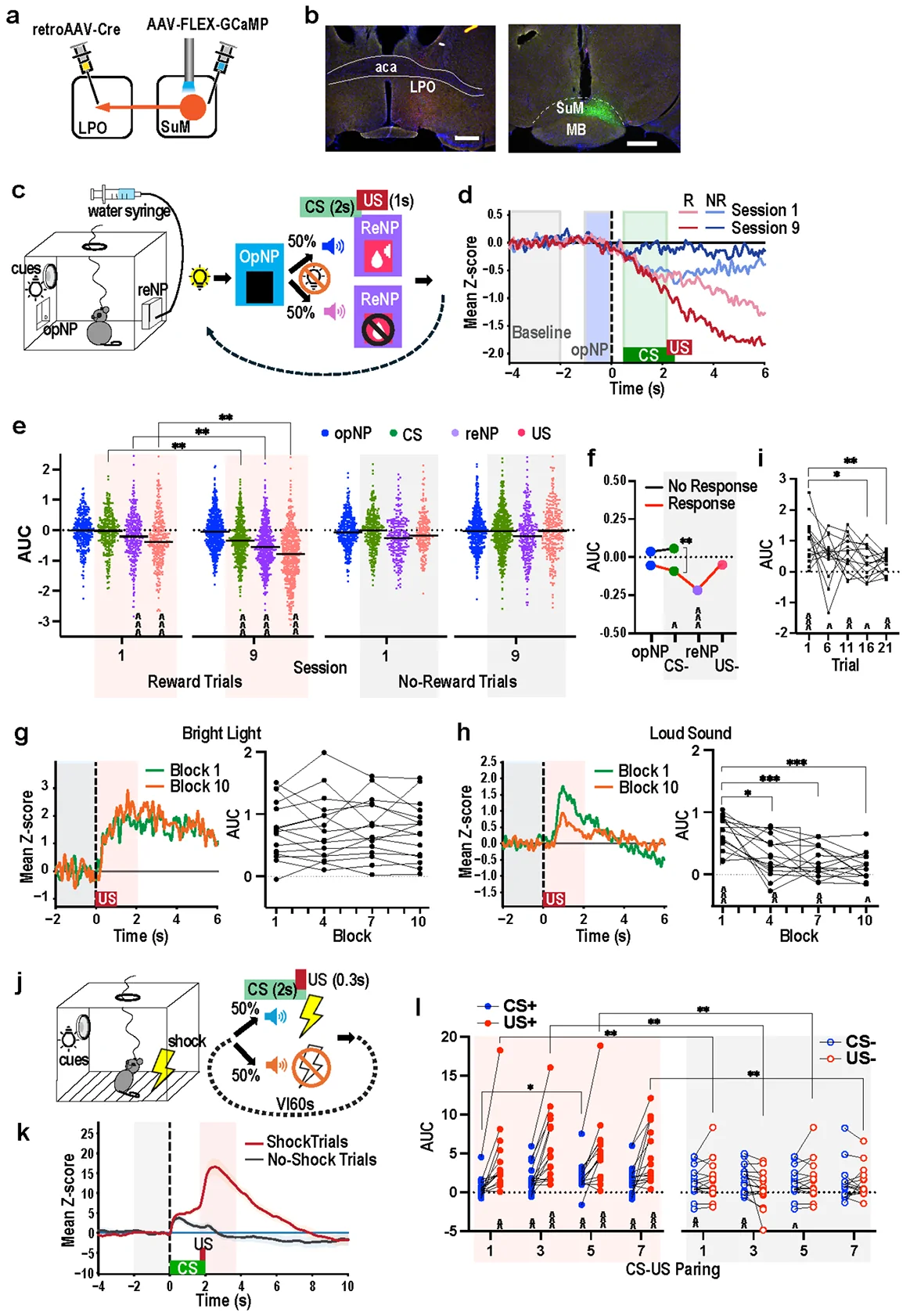
SuM→LPO GCaMP activity during motivated behavior. (a) Viral strategy for SuM→LPO photometry. N = 9 mice. (b) Coronal sections showing LPO injections and SuM fiber placements. Scale bars, 500 μm. (c) Water-seeking task structure. (d) Representative z-scored signals for reward and no-reward trials in sessions 1 and 9, with time 0 is set at the completion of opNP; shaded regions indicate epochs used for AUC analysis. (e–f) AUC values for reward (e) and no-reward (e–f) trials. Šidák tests: ^P < 0.05, ^^P < 0.01, ^^^P < 0.001 vs baseline AUC; **P < 0.01. (g–i) AUC changes following 1-s bright light or loud sound. Šidák tests: ^P < 0.05, ^^P < 0.01, ^^^P < 0.001 vs baseline; *P < 0.05, **P < 0.01, ***P < 0.001. (j) Fear-conditioning schematic. (k) Representative CS-aligned traces for shock and no-shock trials. (l) AUC values for CS and US epochs in sessions 1, 3, 5, 7. Šidák tests: ^P < 0.05, ^^P < 0.01, ^^^P < 0.001 vs baseline; **P < 0.01.

**Figure 4:**
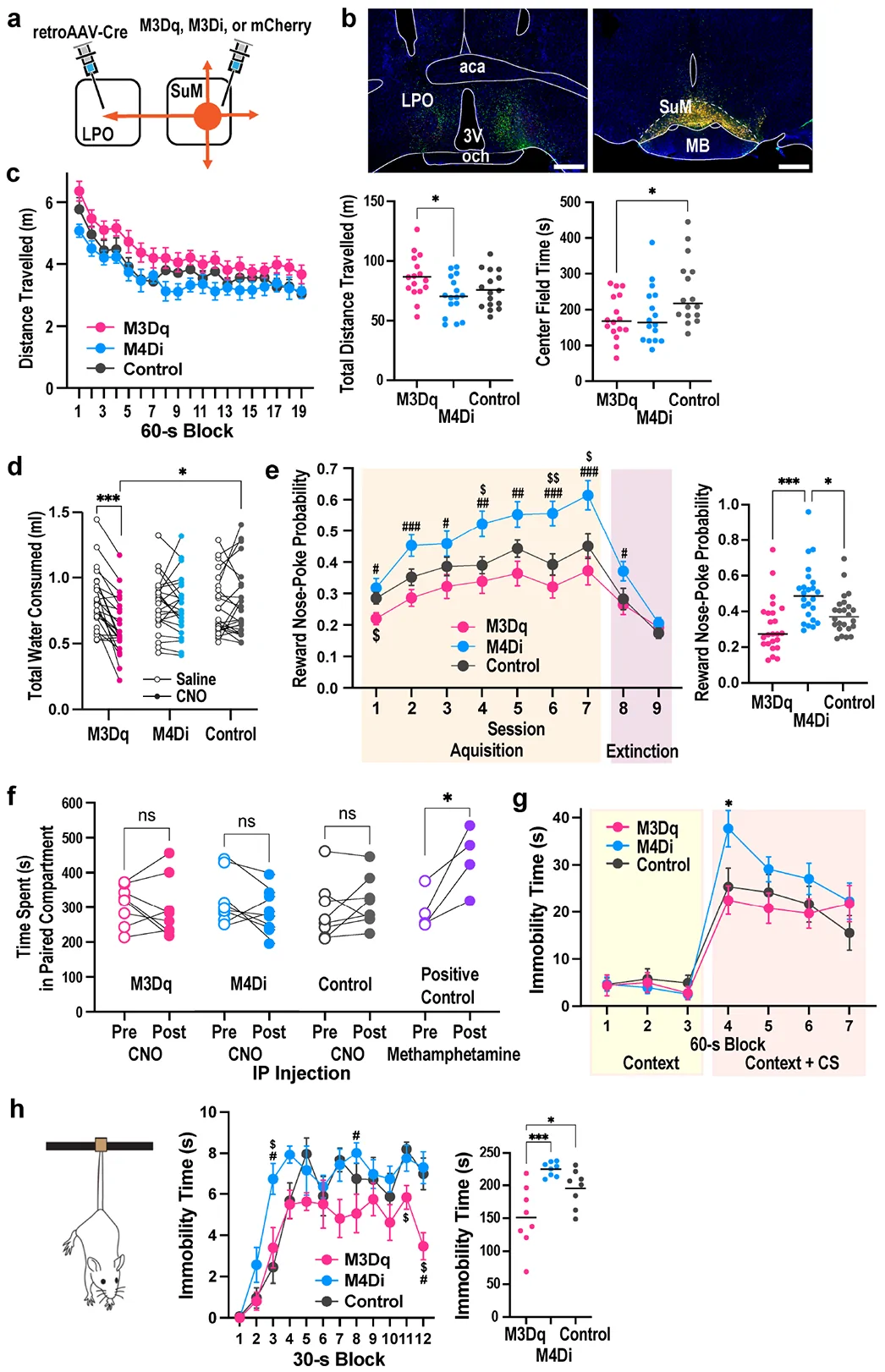
Chemogenetic manipulation of SuM→LPO+Collateral neurons and motivated behavior. (a) Viral strategy for DREADD expression in SuM→LPO+Collateral neurons. (b) Representative labeling in the LPO and SuM. Scale bars, 500 μm. (c) Locomotion and center time in the open field. mCherry: N = 16; M4Di: N = 16; and M3Dq: N = 16. *P < 0.05. (d) Water intake following saline or CNO. mCherry: N = 24; M4Di: N = 24; and M3Dq: N = 24.*P = 0.036; ***P < 0.001. (e) CS-triggered approach probability during acquisition and extinction. mCherry: N = 24; M4Di: N = 24; and M3Dq: N = 24. Šidák tests: $P < 0.05$P < 0.01 vs control; #P < 0.05, ##P < 0.01, ###P < 0.001 vs M4Di; *P < 0.05; ***P < 0.001. (f) Conditioned place-preference scores. mCherry: N = 8; M4Di: N = 8 and M3Dq: N = 8. *P = 0.037. (g) Context and CS-evoked immobility during fear-conditioning test. N = 16; M4Di: N = 16; and M3Dq: N = 16. *P = 0.012 (Tukey’s test). (h) Immobility during tail suspension. mCherry: N = 8; M4Di: N = 8 and M3Dq: N = 8. Šidák tests: $P < 0.05 vs control; #P < 0.05 vs M4Di. Tukey’s test: *P = 0.036; ***P < 0.001.

**Figure 5:**
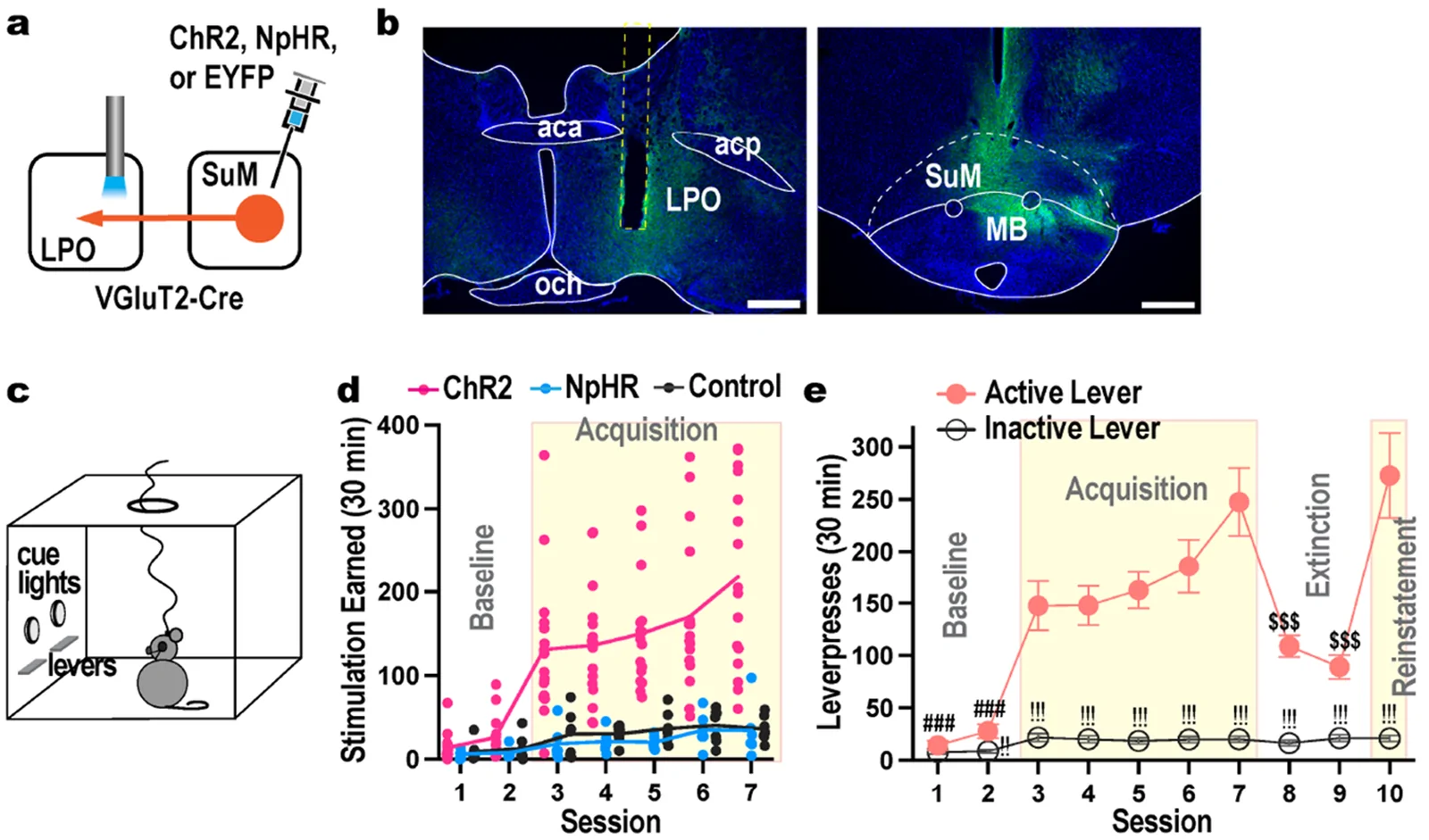
Optogenetic activation of SuM→LPO terminals reinforces approach behavior. (a) Viral injections and fiber placement in VGluT2-Cre mice. (b) Injection sites and fiber trajectories. Scale bars, 500 μm. (c) Intracranial self-stimulation chamber layout. ChR2: N = 16; EYFP: N = 7;andNpHR: N = 6. (d) Photostimulation earned during acquisition (sessions 3–7). ChR2 mice earned significantly more stimulations than EYFP and NpHR mice (P < 0.001). (e) Active and inactive lever presses across acquisition, extinction, and reinstatement. Šidák tests: ***P < 0.001.

**Figure 6:**
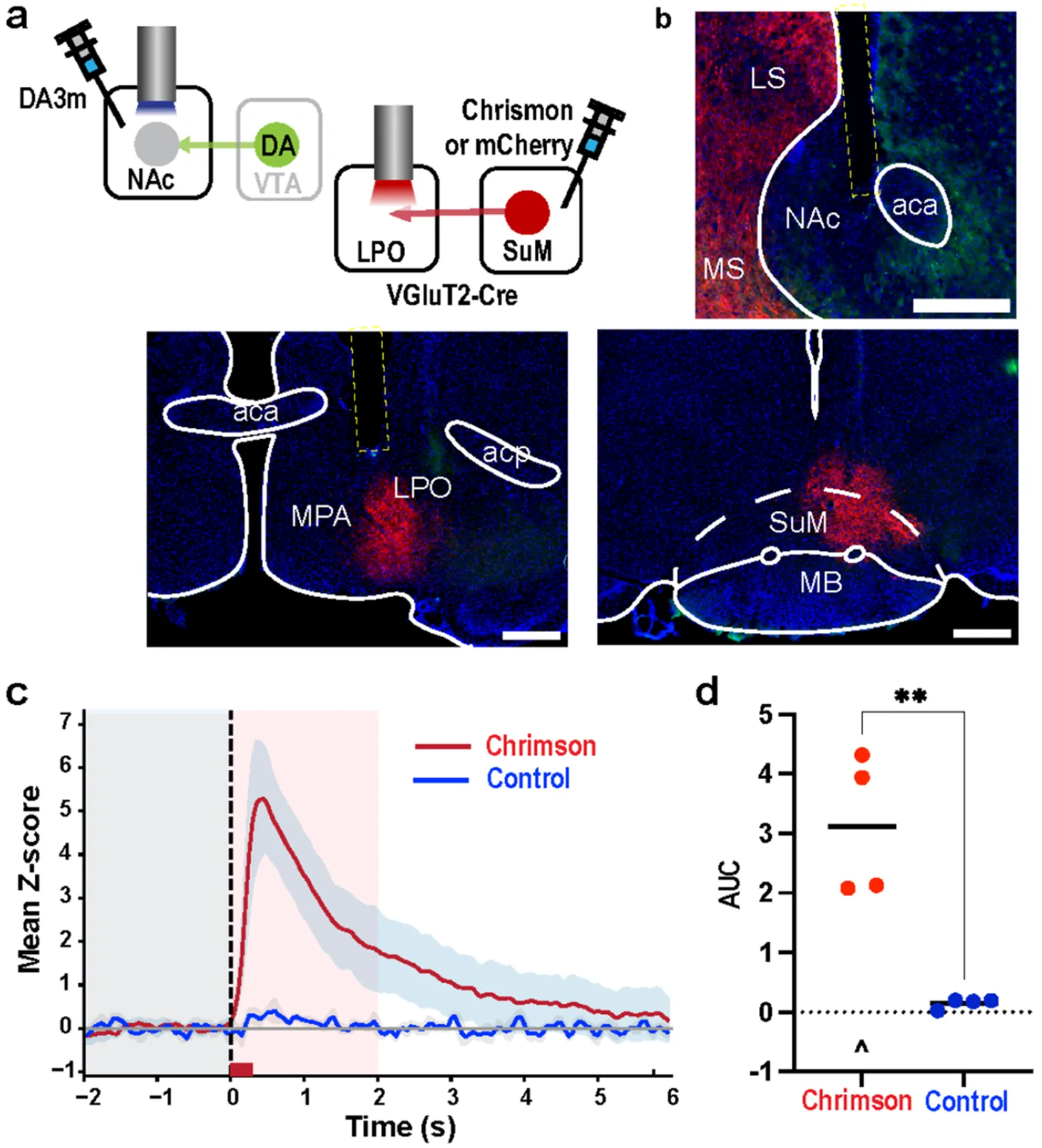
SuM→LPO terminal activation drives NAc dopamine release. (a) Viral injections for DA3m recording and Chrimson (N = 4)/mCherry (N = 4) expression. (b) Chrimson-labeled SuM→LPO terminals (red) and DA3m-expressing NAc neurons (green). Scale bars, 500 μm. (c) DA3m traces aligned to photostimulation onset (8 pulses, 25 Hz). (d) AUC responses in Chrimson and control mice; horizontal bars denote group means. Šidák tests: **P = 0.002; ^P < 0.001 vs baseline.

**Table 1:** Collateral projections of SuM→MS and SuM→LPO

Group	Region	SuM-MS (n = 5)	SuM-LPO (n = 4)
Cortical region	Dorsal tenia tecta	●●●	●●●
	Prelimbic and infralimbic cortex deep layer V/VI	●●	●●
	Cingulate cortex 1 and 2	●	●
	Claustrum	●	●
	CA1 hippocampal field	●●●	●●
	CA2 hippocampal field	●●●	●●
	Dentate gyrus	●●	●●
Basal forebrain	Vertical limb of the diagonal band	●●●	●●●
	Medial septum	●●●	●●●
	Lateral septum	●●	●●●
	Horizontal limb of the diagonal band	●●●	●●●
	Substantia innominata	●●●	●●●
	Ventral pallidum	●●	●●
Thalamus / Epithalamus	Mediodorsal thalamic nucleus	●	●
	Reuniens nucleus	●	●
	Paraventricular thalamus	●	●●
	Lateral thalamus	—	●●
	Lateral habenula	●	●●●
Hypothalamus	Medial preoptic area	●	●●
	Lateral preoptic area	●●●	●●●
	Anterior hypothalamic area	●	●
	Peduncular part of lateral hypothalamus	●●	●●●
	Dorsomedial hypothalamic nucleus	●●	●●
	Ventral tuberomammillary nucleus	●●	●●
Amygdala	Central and medial amygdala nuclei	●	●●
	Amygdalohippocampal area	●	●●
Midbrain / Pons	Medial ventral tegmental area	●	●
	Median raphe nucleus	●●	●●
	Dorsal raphe nucleus	●	●●
	Periaqueductal gray	●	●
	Interpeduncular nucleus	—	●
	Nucleus incertus	●●	●●
	Laterodorsal tegmental nucleus	●●	●●
	Lateral parabrachial nucleus	—	●●

Dots represent projection volume as determined by visual assessment: ●●● indicates large, ●● indicates medium, ● indicates small, and — denotes no detectable projection.

**Table 2:** Procedures and viral vectors

Experiment	Mouse line	Target and AAVs
Anterograde collateral tracing	C57BL/6J	MS or LPO: AAV-retro-EF1a-Cre (200 nl); SuM: AAV1-FLEX-mGFP-2A-SYP-mRuby (100 nl)
Retrograde tracing	C57BL/6J	LPO: 1% Fluoro-Gold (100 nl); MS: 0.5% CTB (150 nl)
SuM→LPO photometry during salient stimuli	C57BL/6J	LPO: AAV-retro-EF1a-Cre (200 nl); SuM: AAV9-Syn- FLEX -GCaMP7s (100–200 nl)
Chemogenetic manipulation	C57BL/6J	LPO: AAV-retro-EF1a-Cre (200 nl); SuM: AAV9-hSyn-DIO-hM4D(Gi)-mCherry; AAV9-hSyn-DIO-hM3D(Gq)-mCherry; or AAV9-hSyn-DIO-mCherry (100–200 nl)
Optogenetic ICSS	VGluT2-Cre	SuM: AAV9-EF1a-DIO-ChR2-EYFP, AAV9-EF1a-DIO-eNpHR3.0-EYFP, or AAV9-EF1a-DIO- EYFP (100 nl)
NAc DA3m during SuM→LPO optogenetic stimulation	VGluT2-Cre	SuM: AAV5-Syn-FLEX-rc[ChrimsonR-tdTomato] and AAV9-hSyn-DIO-mCherry (100 nl); NAc: AAV5-hSyn-GRAB-DA3m (200 nl)

**Table 3:** Stereotaxic coordinates

Brain region	AP	ML	DV	Angle
MS	+ 0.8	0	−4.0	0°
LPO	0	+ 0.9 or ± 0.9	−5.1	0°
SuM	−2.7	+ 0.2 or 0	−4.6	0°
NAc	+ 1.1	+ 0.8	−4.5	0°

**Table 4:** Experimental Designs and Statistical Tests (SPSS v31.0.1.0 or GraphPad Prism v.11.02)

Experiments	DV	Test	Factor Name	N
Figure 2d: RNAscope	Labeled cell count	One-way within-subjects ANOVA	W: Receptor type (OX1R, OX2R, double)	4 mice
Figure 3e: Water-Seeking Task-Rewarded	GCaMP signal AUC	Two-way mixed ANOVA	W: Epoch (baseline, opNP, CS, reNP, US) B: Day (1, 9)	Day 1: 297 trials Day 9: 496 trials from 9 mice
Figure 3f: Water-Seeking Task-Not Rewarded	GCaMP signal AUC	Two-way mixed ANOVA	W: Epoch (baseline, opNP, CS) B: Day (1, 9)	Day 1: 297 trials Day 9: 496 trials from 9 mice
	GCaMP signal AUC	Two-way mixed ANOVA	W: Epoch (baseline, reNP, US) B: Day (1, 9)	Day 1: 215 trials Day 9: 272 trials from 9 mice
Figure 3g: Bright Light Tests	GCaMP signal AUC	Two-way within-subjects ANOVA	W: Epoch (baseline, light) W: Block (1, 4, 7, 10)	16 mice
Figure 3h: Loud Noise Tests	GCaMP signal AUC	Two-way within-subjects ANOVA	W: Epoch (baseline, light) W: Block (1, 4, 7, 10)	15 mice
Figure 3i: Loud Noise Tests	GCaMP signal AUC	Two-way within-subjects ANOVA	W: Epoch (baseline, sound) W: Trial (1, 6, 11, 16)	15 mice
Figure 3l: Fear Conditioning	GCaMP signal AUC	Three-way within-subjects ANOVA	W: Context (shock, no_shock) W: Epoch (baseline, CS, US) W: Trial (1, 3, 5, 7)	16 mice	
Figure 4c: Open-field Test	Locomotor distance	Two-way mixed ANOVA	W: Block (1–19) B: Group (M3Dq, M4Di, control)	16 mice per group
	Center-field time	Two-way mixed ANOVA	W: Block (1–19) B: Group (M3Dq, M4Di, control)	16 mice per group
Figure 4d: Water Intake Test	Water consumed	Two-way mixed ANOVA	W: Treatment (saline, CNO) B: Group (M3Dq, M4Di, control)	24 mice per group
Figure 4e: Approach for Water	Probability for approach response	Two-way mixed ANOVA	W: Session (1–7) B: Group (M3Dq, M4Di, control)	24 mice per group
Figure 4f: Conditioned Place Preference	Time spent	T-tests	W: Treatment (before, after)	8 mice per group
Figure 4g: Fear Conditioning	Immobility time	Two-way mixed ANOVA	W: Block (1–7) B: Group (M3Dq, M4Di, control)	16 mice per group
Figure 4h: Tail Suspension Test	Immobility time	Two-way mixed ANOVA	W: Block (1–12) B: Group (M3Dq, M4Di, control)	8 mice per group
Figure 5d: ICSS	Stimulation # earned	Two-way mixed ANOVA	W: Session (3–7) B: Group (ChR2, NpHR, control)	ChR2: N = 16; EYFP: N = 7; NpHR: N = 6
Figure 5e: ICSS	Lever presses	Two-way mixed ANOVA	W: Session (1–10) B: Lever (active, inactive)	16

Abbreviations: B, between factor; DV, dependent variable; W, Within factor Note: GCaMP signal data from all trials of individual mice were pooled and analyzed using the described methods.
